# Residues of veterinary drugs and heavy metals in bovine meat from Urabá (Antioquia, Colombia), a promising step forward towards international commercialization

**DOI:** 10.1016/j.vas.2021.100192

**Published:** 2021-08-04

**Authors:** Diego Alonso Restrepo Molina, Jairo Humberto López Vargas, Jesús Alfredo Berdugo Gutierrez, Andrés Gallo-Ortiz, Yudy Duarte-Correa

**Affiliations:** a Department of Agricultural and Food Engineering, Faculty of Agricultural Sciences, Universidad Nacional de Colombia, 050034, Medellín, Colombia; b INTAL Research Group. INTAL Foundation, Institute of Food Science and Technology, 055411, Itagüí, Colombia; c BIOALI Research Group, Food Department, Faculty of Pharmaceutical and Food Sciences, Universidad de Antioquia, 050010, Medellín, Colombia

**Keywords:** Meat quality, Bovines, Chemical contamination, Veterinary drugs, Heavy metal contamination, Food safety

## Abstract

Veterinary drugs are used for disease control in bovines Their presence at acceptable levels is a cause of concern for consumers and control agencies, as well as being a limitation for accessing international markets. The objective of this research was to evaluate the presence of residues of veterinary drugs and heavy metals in meat cuts from the Urabá region in Colombia. From a total of 80 samples of meat cuts from the loin and neck, we determined the presence of 29 veterinary drug residues and of cadmium and lead. The drug residues were quantified by liquid chromatography coupled to mass spectrometry. Heavy metals were measured by electrothermal atomic absorption. As a result, all evaluated samples complied with Colombian and European regulations for drug residues and heavy metals. These results demonstrate good veterinary practices used for bovines raised in this part of Colombia, and they represent an export opportunity and an opening for new markets that can be enhanced by the implementation of a meat quality seal from this region.

## Highlights

• Residues of veterinary drugs in samples of beef from Urabá (Antioquia, Colombia) were found below the maximum limits allowed by national and international regulations (European Union).
• Cadmium and lead content in samples of bovine cuts from Urabá (Antioquia, Colombia) were found below the maximum permissible limits of national and international regulation (European Union).
• There were no differences in the loin and neck cut meat samples, fulfilling the applicable legal requirements for both cases.

## Graphical abstract

**Figure:**
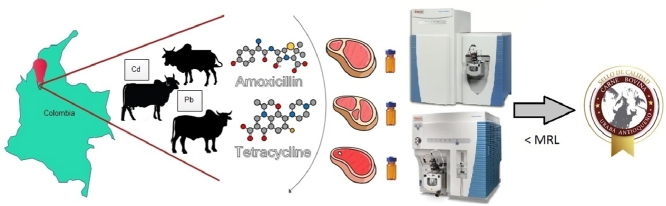

## 1 Introduction

Colombian geography promotes the production of a great variety of foods such as bananas, avocado, passion fruit, coffee, sugar cane, cocoa, mango, milk, and dairy products as well as meats and their derivatives. Many of these agricultural products are traded internationally (Peñuela Mesa et al., 2013). For beef, Antioquia is the leading department in number of cattle with more than three million and about 2.7 million hectares of natural pasture both improved and natural forage, as well as silvopastoral systems for producing milk, meat, or both (FEDEGAN, 2021). Specifically, in the Urabá region, an area of 11,664 km^2^ of territory includes 11 municipalities of the department, recognized mainly for the production and commercialization of bananas. However, cattle also boast important production and is an important activity relevant to the local economy. Cattle production is ranked very high compared to other regions of the department, and has advantages such as herds being certified and free of foot-and-mouth disease and certified and enabled for refrigeration by current national regulations, enabled by a high capacity for refrigeration and slaughter. Meat processing companies in the Urabá region are market leaders at the national level (Figueroa-Delgado, 2019). Though Colombia has ideal topographic conditions, high-quality animal genetics, and production systems that place the country as the third-largest meat producer in Latin America (PROCOLOMBIA, 2016), this is an economic sector that still needs to be revitalized since currently, cattle do not represent a large share in country exports.

In the international market, contaminants in food products of animal origin constitute risk factors for health and are one of the greatest barriers to commercialization (Stewart et al., 2002). Meat is considered a food with a high risk of contamination since it can be an important route of exposure to potentially toxic agents such as residues of veterinary drugs and pesticides, mycotoxins, heavy metals, nitrites, benzopyrene, dioxins, and furans, etc. (Beyene, 2015; Nichea et al., 2015). The presence of these substances in meat is usually associated with inadequate agricultural or livestock practices, lack of controls in transport and storage, and deficiency in the handling and preparation of the product (Richi et al., 2015). The use of veterinary drugs in livestock production has been associated with therapeutic processes for the management of infections or non-contagious diseases and on some occasions as growth promoters. The distinction between therapeutic use and growth promotion is that the first one alleviates pain and suffering and the second one aims to increase weight gain and improved feeding efficiency to achieve market weight in less time than if the antimicrobials were not in the feed (Baynes et al., 2016). According to the *Codex Alimentarius*, veterinary drugs can be classified by their mode of action as antibacterial, antimicrobial, antiparasitic, antiprotozoal, fungicide, anthelmintic, beta-blocker, glucocortysteroid, insecticide, growth promoter, tranquilizer, and trypanosomicide (FAO, 2018). However, as growth promoters, there is potential for the emergence of antimicrobial resistance in human pathogenic bacteria as a result of the administration of antimicrobial growth promoters. This can potentially compromise the therapeutic effectiveness of antimicrobial agents in veterinary and human medicines (Brown et al., 2017). As a consequence of widespread public health concerns, Sweden banned the use of all growth-promoting agents in 1986 and this change has been implemented throughout the European Union (EU) (Brown et al., 2017). In general, the irrational use of veterinary drugs and the failure of strict adherence to withdrawal and withholding time for the drugs can cause adverse effects such as the development of resistance and residual effects in the animals (Ture et al., 2019). Potential adverse health effects in humans, when exposed to residues of some veterinary drugs used in food-producing animals can include allergic reactions to several antimicrobial drug classes ranging from minor reactions like skin rashes to severe anaphylaxis, blood dyscrasias (including hemolytic anemia, neutropenia, thrombocytopenia, and pancytopenia), disruption of normal intestinal human flora, hepatitis, carcinogenicity, cardiovascular toxicity, and development of antimicrobial resistance that make it difficult to treat human infections (Baynes et al., 2016). Specific effects include allergic reactions caused by residues of penicillin, sulfonamides, and streptomycin (Lozano & Arias, 2008). Effects also include increased bacterial resistance caused by aminoglycosides, beta-lactams, tetracyclines, and sulfonamides (Jana & Deb, 2006), ototoxicity, and nephrotoxicity caused by aminoglycosides, skin reactions, severe toxidermia and epidermal toxic necrolysis caused by sulfonamides (Baynes et al., 2016). Therefore, their presence above acceptable levels is a matter of concern for both the surveillance and control agencies and consumers.

Heavy metals are dispersed in the environment through industrial effluents, organic wastes, refuse burning, and transport and power generation; and they can be carried to places many miles away from the sources by wind, onto the land, or the surface of waterways (Mahurpawar, 2015). They are high-density chemical elements that can become toxic with excessive intake, and they can even cause death. Their presence in foods of animal origin is associated with inadequate agricultural or livestock practices (Bustamante et al., 2015). Their incorporation occurs through the diet of animals since they are bioaccumulated in muscle tissues and viscera (Rudy, 2010). Toxicosis is the term used to describe the syndrome of adverse health effects that result from exposure to a toxicant (Gupta, 2018). Among the signs and symptoms of toxicosis on human health, heavy metals can cause central and peripheral neuropathies, ulcers perforation of the nasal septum, respiratory cancer, proteinuria, osteomalacia, aminoaciduria, emphysema, central nervous disorders, and anemia. They can also affect the ability to smell and decrease fertilization rates (Mahurpawar, 2015). The nature of the toxic responses depends not only on the toxicant but also the route of exposure, the duration, and intensity of the exposure, and the characteristics of the exposed individual, i.e. species, gender, age, pre-existing disease states, nutritional status, and prior exposure to the agent or related compounds (Gupta, 2018). Animals and humans may be exposed through multiple routes, including oral, dermal, and pulmonary.

Specifically, lead and cadmium are ubiquitous heavy metals in the environment with food, water, air, tobacco smoke, and alcoholic drinks as possible sources of exposure (Kresovich et al., 2015). Lead and cadmium have both been associated with testicular toxicity and impaired fertility in several species (Gupta & Wong Wai Leong, 2011). Lead also appears to be able to adversely affect the ability of spermatozoa to fertilize ova; but this effect, like others associated with lead exposure, appears to be dependent on age and individual variations in susceptibility, adaptation, and reversibility (Sokol, 2006). Like lead, cadmium adversely affects male reproduction and spermatogenesis when the stage of the seminiferous epithelium associated with the production of sperm is specifically inhibited by cadmium (Gupta & Wong Wai Leong, 2011). Cadmium can also interfere with the cellular metabolism of zinc, an essential trace element necessary for normal reproduction. Diets deficient in zinc can predispose individuals to the toxic effects of cadmium (Akinloye et al., 2006). Considering the importance and health effects of these toxic substances, this research aimed to determine the compliance of the applicable regulations relating to the presence of veterinary drug residues and heavy metals in meat cuts from the Urabá region in the Department of Antioquia, Colombia, leading to the establishment of an Antioquia Quality Seal for Bovine Meat Cuts, that seeks to deliver high-quality products to new markets.

## 2 Materials and methods

### 2.1 Study area

**Fig. 1:**
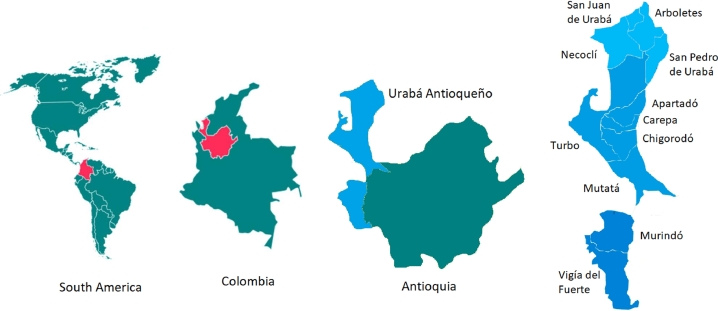
Geographic location of the study zone.

This study was carried out in the Urabá region of the Department of Antioquia, Colombia, (25 °C - 31 °C, 85% relative humidity, pluviosity 100-300 mm/month). The sampling was carried out in 10 of the 11 municipalities that make up the area: *Arboletes, San Juan de Urabá, San Pedro de Urabá, Necoclí, Apartadó, Carepa, Chigorodó, Turbo, Mutatá and Murindó* (Fig. 1).

Initially, 55 farms with exclusive livestock activities were enrolled in the study and had cattle herds registered by the Colombian Agricultural Institute (ICA). These farms all had production systems and similar veterinary and agronomic management, improved pastures, rotational grazing, with veterinary advice and vaccination records against leptospira, brucellosis, blackleg, and to a lesser extent bovine rabies, and without the use of hormonal implants. An observational study was carried out to determine the quality of the meat from the forequarters of cattle from the Urabá region. The animals were not slaughtered for experimental purposes, the cattle were enrolled under current local regulations and the meat samples (neck and loin) were measured separately. They were identified according to the place of origin of the animal.

### 2.2 Samples collection

**Fig. 2:**
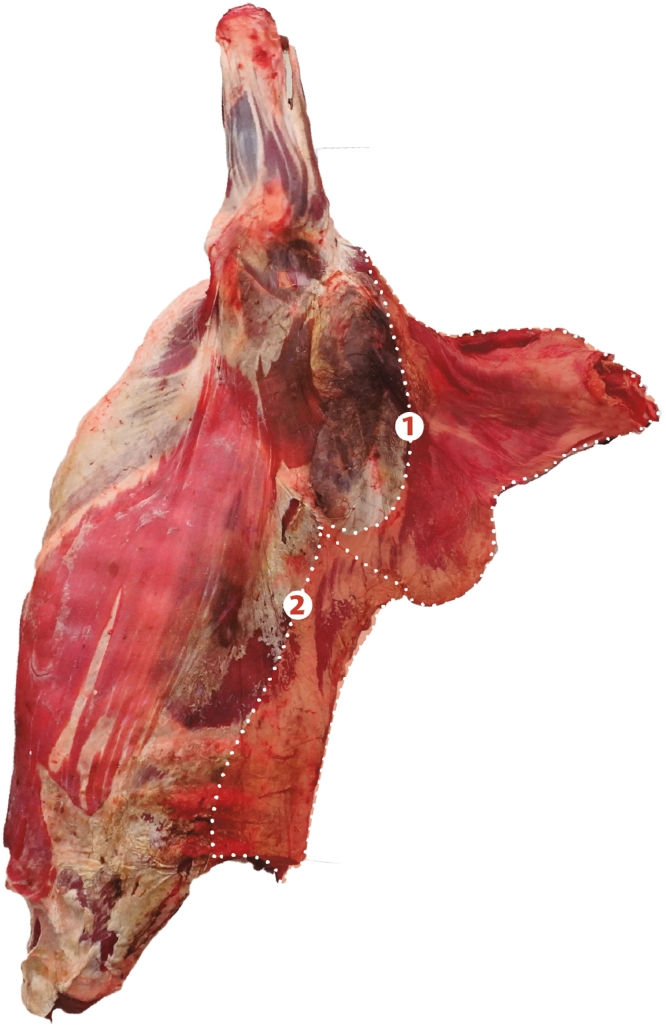
Meat cuts obtained from the forequarter: neck (1) and loin (2).

Once the place of origin of the animal was identified, sampling was carried out randomly from chosen meat markets in the different towns of the Urabá region; and the following number of samples were collected by municipality, *Arboletes* (12), *San Juan de Urabá* (8), *San Pedro de Urabá* (10), *Necoclí* (10), *Apartadó* (12), *Carepa* (8), *Chigorodó* (6), *Turbo* (5), *Mutatá* (6) and *Murindó* (3). The meat included samples from castrated males of the Zebu breeds and its crosses ranging in age from between 2 and 4 years, and that was maintained grazing in pastures of brachiaria (*Brachiaria humidicola*) with minerals, and water *ad libitum*. Muscle tissue samples corresponding to the loin (40 samples) and neck (40 samples) were taken. Fig. 2 shows the location of the cuts in a carcass, and a more detailed description of the cut is presented as supplementary information S1.

Samples were vacuum packed and transferred under freezing conditions (-20 °C) to the laboratory where they were homogenized in a meat mill (EG-22-A1 Model, Torrey) and stored for a maximum of eight (8) days until subsequent analysis. Although tissues with the highest heavy metals are usually liver, kidney, or spleen, with residues in meat (muscle) being lower (Moreno & Lanusse, 2017), this study focused to determine only the quality of the meat of the bovine fore-train of animals from the Urabá region of Antioquia.

### 2.3 Determination of veterinary drugs by liquid chromatography coupled with high-resolution mass spectrometry (LC-HRMS)

The analyzed veterinary drugs are listed in Table 1, which listed the operation conditions, limits of detection (LOD), and limits of quantification (LOQ) of the methods used. LOD and LOQ are defined as the lowest concentration of the analyte that can be reliably detected and quantified. The analyses were carried out as proposed by Berendsen et al. (2017) with some modifications. For the preparation of the material, 2.0 g of homogenized sample were weighed in a polypropylene tube (50 ml), 10 ml of acetonitrile/methanol/water (7:1:2) were added and stirred for 15 min. Subsequently, this mixture was subjected to an ultrasonic bath (Elmasonic P, Elma) for 10 min, and QuEChERS Vet Drug Pouch (Agilent Technologies, United States) was added. The samples were agitated for 3.0 min and centrifuged at 4500 rpm for 5 min, and the supernatant was analyzed immediately.

**Table 1:** Analyzed veterinary drugs.

**Class**	**Name**	**Abbr.**	**LC - MS**	**Ionization mode**	**Average experimental mass (m/z)**	**Precursor ion (m/z)**	**Product ion (m/z)**	**Collision energy (eV)**	**Retention time (min)**	**LOD(µg/kg)**	**LOQ(µg/kg)**	Colombian Limit(Muscle)µg/kg*	EU Limit(Muscle)µg/kg**
Antimicrobial Agent	Amoxicillin	AMOX	LC-HRMS	[M+H]^+^	366.11237	N.A			0.55	1.5	5.0	50	50
	Benzylpenicillin / Procaine Benzylpenicillin	DCI	LC-HRMS	[M+H]^+^	335.10632	N.A			5.41	15	50	50	50
	Ceptiofur	CEPT	LC-HRMS	[M+H]^+^	524.03629	N.A			4.11	1.5	5.0	1000	1000
	Ciprofloxacin	CPFN	LC-HRMS	[M+H]^+^	332.14078	N.A			0.59	10	30	NA	100
	Tilmicosin	TCN	LC-HRMS	[M+H]^+^	869.57251	N.A			7.23	0.8	2.5	100	50
	Tylosin	TLS	LC-HRMS	[M+H]^+^	916.52734	N.A			7.66	0.8	2.5	100	100
	Chlortetracycline	CTCN	LC-HRMS	[M+H]^+^	479.12305	N.A			6.15	1.5	5.0	200	100
	Oxytetracycline	OTCN	LC-HRMS	[M+H]^+^	461.15546	N.A			5.15	1.5	5.0	200	100
	Tetracycline	TCC	LC-HRMS	[M+H]^+^	445.16107	N.A			5.40	1.5	5.0	200	100
	Colisti	CLT	LC-HRMS	[M+H]^+^	289.69540	N.A			7.51	15	50	150	150
	Danofloxacin	DFC	LC-HRMS	[M+H]^+^	358.15652	N.A			5.67	0.8	2.50	200	200
	Spectinomycin	EPMC	LC-HRMS	[M+H]^+^	333.16595	N.A			0.54	15	50	500	300
	Spiramycin	ESPM	LC-HRMS	[M+H]^+^	843.52128	N.A			0.54	10	30	200	200
	Dehydrostreptomycin	D-ETPM	LC-HRMS	[M+H]^+^	584.28918	N.A			0.53	15	50	600	500
	Streptomycin	ETPM	LC-HRMS	[M+H]^+^	582.27393	N.A			0.49	15	50	600	500
	Monensin	MNS	LC-HRMS	[M+H]^+^	669.42310	N.A			11.58	1.5	5.0	10	2
	Neomycin	NEOM	LC-HRMS	[M-H]^−^	669.42310	N.A			11.47	45	150	500	500
	Flumequine	FQN	LC-HRMS	[M+H]^+^	262.08755	N.A			8.40	0.8	2.5	500	200
	Enrofloxacin	EFX	LC-HRMS	[M+H]^+^	360.17180	N.A			5.70	1.5	5.0	NA	100
	Florphenicol	FFC	LC-HRMS	[M+H]^+^	358.00774	N.A			6.52	10	30	NA	200
Antiparasitic Agent	Diazinon	DZN	LC-MS/MS	[M+H]^+^	N.A	305.094	96.910	6	20	6	20	NA	20
	Fenvalerate	FVT	LC-MS/MS	[M+NH_4_]^+^	N.A	437.132	125.000	3	10	3	10	NA	25
	Permethrin	PMT	LC-MS/MS	[M+NH_4_]^+^	N.A	408.149	153.000	3	10	3	10	50	50
	Thiabendazole	TBZ	LC-MS/MS	[M+H]^+^	N.A	202.019	130.960	6	20	6	20	100	100
Antiprotozoal Agent	Imidocarb	IDC	LC-HRMS	[M+H]^+^	366.20368	N.A			12.95	15	50	300	300
Insecticide	Cyfluthrin	CFT	LC-MS/MS	[M+NH_4_]^+^	N.A	451.142	127.000	6	20	6	20	20	10
	Cyhalothrin	CHT	LC-MS/MS	[M+NH_4_]^+^	N.A	467.088	141.000	6	20	6	20	20	NA
	Cypermethrin	CPMT	LC-MS/MS	[M+NH_4_]^+^	N.A	433.139	127.000	6	20	6	20	50	20
	Deltamethrin	DTMT	LC-MS/MS	[M+NH_4_]^+^	N.A	523.099	281.000	6	20	6	20	30	10
	Fluazuron	FZN	LC-HRMS	[M+H]^+^	506.00921	N.A			11.82	6	20	200	200
Adrenoceptor antagonist	Clenbuterol	CBT	LC-HRMS	[M+H]^+^	277.08719	N.A			5.88	0.03	0.10	0.2	0.1

*Resolution 1382 (2013) (Ministerio de Salud y Protección Social, 2013)

** Reg. EU. 37/2010 (EU., 2010)

Separation was performed by an LC Ultimate 3000 UHPLC (Thermo Fisher Scientific) on a Raptor C18 100 × 2.1 mm, 2.7 µm analytical column (Restek) by gradient elution at a flow rate of 400 µl/min. Elution solvents were solvent A (0.1% formic acid in water) and solvent B (0.1% formic acid in acetonitrile). The gradient was programmed as follows: 0 min 5% B, 0-1 min 5% B, 1-5.5 min 35% B, 5.5-8 min 55% B, 8-9 min 90% B, 9-11 min 90% B, and 11-11.5 90.5% B with a total time of 15 min.

The LC system was coupled to a high-resolution Q-Exactive Orbitrap mass analyzer (Thermo Fisher Scientific) equipped with a heated electrospray ionization interface (HESI). The HRMS was operated in the t-SIM experiment (isolation window m/z:2) to filter the molecular adducts by the quadruple according to Table 1 and with the following parameters (positive and negative ion mode), capillary temperature 300 °C, S-lens RF level 55.0, spray voltage 4.0 Kv, AGC target value 1e6, sheath gas (N_2_) flow rate 50 arbitrary units (a.u), auxiliary gas (N_2_) flow rate 13 a.u. The scanning range of the masses was 150–1500 m/z. The resolution was set at 70,000. The average mass measurement error was less than 5 ppm. Xcalibur 4.0 software (Thermo Fisher Scientific) was used.

### 2.4 Determination of veterinary drugs by liquid chromatography coupled to triple-quadrupole mass spectrometer (LC-MS/MS)

The analyzed veterinary drugs are listed in Table 1, with the LOD and LOQ of the methods used. The analyses were carried out following the methodology proposed by Oliveira et al. (2018) with some modifications. For the preparation of the material, 5.0 g of the sample were weighed and mixed for 1.0 min with 10 ml of water and 10 ml of acetonitrile with 1.0% (v/v) of acetic acid. Subsequently, a vial with QuEChERS (Agilent Technologies) was added and the samples were agitated for 2.0 min and centrifuged (5 min, 4000 rpm). Then 1.0 ml of the supernatant was taken and added to a dispersive cleaning tube (SPE 2 ml, Fatty Samples, Agilent Technologies), stirred for 2.0 min, and centrifuged (5 min, 4000 rpm). Finally, 500 µl of the supernatant was added to an amber vial with 500 µl of acetonitrile. Then, 10 µl of triphenylphosphate (100 µg/ml) was added as an internal standard (Reg. EU. 37/2010).

Separation was performed by an LC Ultimate 3000 UHPLC (Thermo Fisher Scientific) with a Hypersil GOLD aQ C18 analytical column (100 × 2.1 mm, 1.9 µm) (Thermo Fisher Scientific) by gradient elution at a flow rate of 400 µl/min. The elution solvents were solvent A (5 mM ammonium formate and 0.1% formic acid in water:methanol in the ratio 95:5 v/v) and solvent B (5 mM ammonium formate and 0.1% of formic acid in methanol:water in the ratio 95:5 v/v). The gradient was programmed as follows: 0 min 0% B, 0-2 min 5% B, 2-12 min 50% B, 12-15 min 90% B, 15-15.5 min 100% B with a total time of 19 min. The UHPLC system was coupled to a TSQ-Vantage triple quadrupole mass analyzer (Thermo Fisher Scientific) equipped with a HESI. The monitor was operated in positive ionization selective reaction monitoring (SRM) mode. The MS operating conditions were as follows: spray voltage 4500 V, S-lens RF level 55.0, capillary temperature 200 °C, vaporizer temperature 300 °C, sheath gas pressure 60 a.u, auxiliary gas pressure 15 a.u, ion sweep gas pressure 2.0 a.u, and argon (collision gas) pressure 1.5 mTorr. Specific parameters for each compound are listed in Table 1. Xcalibur 2.2 software (Thermo Fisher Scientific) was used.

### 2.5 Determination of heavy metals Cd and Pb was accomplished by graphite furnace atomic absorption spectrophotometry (GFAAS)

Lead and cadmium content was determined using microwave-assisted digestion and graphite furnace atomic absorption spectrophotometry (GFAAS) according to the Official Method 999.10. Lead, cadmium, zinc, copper, and iron were determined by the method Foods. Atomic Absorption Spectrophotometry after Microwave Digestion (AOAC, 2010). Digestion was carried out in a microwave digester (Ethos One digester, Milestone). A portion of 0.5 g of each sample was weighed on each PTFE container, 1.0 ml of hydrogen peroxide at 30% (w/w) and 7.0 ml of nitric acid at 65% (w/w) were added. The equipment started at 25 °C and reached 180 °C in 15 min. Then it was maintained at that temperature for 15 min at 1600 W. Subsequently, the samples were cooled, and the volume was completed to 25 ml with deionized water. Finally, the samples were analyzed in an iCE 3000 Series graphite furnace atomic absorption spectrophotometer (Thermo Fisher Scientific). LOD and LOQ for the methods used were 0.003 mg/kg and 0.010 mg/kg for lead, and 0.003 mg/kg and 0.010 mg/kg for cadmium.

### 2.6 Statistical analysis

Results for veterinary drugs and heavy metals were expressed as the average of three measurements. An analysis of variance (ANOVA) with Fisher's least significant difference (LSD) test was used to determine the significant differences between the means at a significance level of *p* < 0.05 using the Statgraphics Centurion XVI software (Statistical Graphics Corporation, Ver. 16.0.07).

## 3 Results and discussion

### 3.1 Residues of veterinary drugs

The results of the veterinary drug residue analysis are listed in Table 2. There were no quantifiable levels of the analyzed compounds in any of the evaluated factors. All factors were under the limit of quantification (LOQ) of the method used for veterinary drug residues. Therefore, in the enrolled municipalities the meat cuts comply with Colombian regulations: Resolution 1382 (2013) (Ministerio de Salud y Protección Social, 2013) and even with the applicable European Union regulations: Reg. EU. 37/2010 (EU., 2010), on the maximum residue limits (MRL) of veterinary drugs in foodstuffs of animal origin. According to the European Medicines Agency (EMA), the MRL is the maximum allowed concentration of residue in a food product obtained from an animal that has not received veterinary medicine or that has been exposed to a biocidal product for use in animal husbandry (EMA, 2018).

**Table 2:** Veterinary drug residue content in meat samples obtained from cattle raised in the Urabá region (Antioquia).

**Municipality**	**Number of Samples**	**NEOM**	**DCI, EPMC, CLT, IDC, ETPM,D-ETPM**	**ESPM, FFC, CPFN**	**CFT, CHT, CPMT, DTMT, TBZ, DZN, FZN**	**FVT, PMT**	**AMOX, MNS, TCC, CTCN, DCI, OTCN, EFX, CEPT**	**FQN, DFC, TCN, TLS**	**CBT**
*Arboletes*	12	˂150	< 50	< 30	<20	< 10	˂5	˂2.5	< 0.10
*San Juan de Urabá*	10	˂150	< 50	< 30	<20	< 10	˂5	˂2.5	< 0.10
*Necoclí*	8	˂150	< 50	< 30	<20	< 10	˂5	˂2.5	< 0.10
*San Pedro de Urabá*	9	˂150	< 50	< 30	<20	< 10	˂5	˂2.5	< 0.10
*Apartadó*	12	˂150	< 50	< 30	<20	< 10	˂5	˂2.5	< 0.10
*Carepa*	8	˂150	< 50	< 30	<20	< 10	˂5	˂2.5	< 0.10
*Chigorodó*	6	˂150	< 50	< 30	<20	< 10	˂5	˂2.5	< 0.10
*Turbo*	5	˂150	< 50	< 30	<20	< 10	˂5	˂2.5	< 0.10
*Mutatá*	2	˂150	< 50	< 30	<20	< 10	˂5	˂2.5	< 0.10
*Murindó*	3	˂150	< 50	< 30	<20	< 10	˂5	˂2.5	< 0.10

The results are expressed in µg/kg.

Since in the participating municipalities the meat cuts complied with Colombian and EU regulations, we assumed that the meat production scheme in the Urabá region has a well-controlled veterinary drug management system, showing the effectiveness of the controls carried out by the regulatory agencies.

In general, the use of products of veterinary medicine in livestock production has been associated with therapeutic processes for the management of infections or diseases. If they are used correctly, they allow excellent and abundant food production from animal origins (Moreno & Lanusse, 2017). Therefore, these results added to the positive characteristics of the region, and they represented a great opportunity for the addition of meat products into international markets. Other researchers have determined contaminants in meat, shown in many cases by the presence of trace components in food samples (Casado et al., 2016), and relating them to acute or chronic toxicity, mutagenic, and carcinogenic effects (Lozano & Arias, 2008). Segura Castro et al. (2017) identified the presence of oxytetracycline residues (OTCN) in 81 samples of fresh beef marketed in Córdoba (Colombia). They used HPLC for quantification and found that 4.9% of the samples showed detectable levels that did not exceed the legal limits. Casado et al. (2016) evaluated the presence of 23 drug residues in beef marketed in supermarkets in Madrid (Spain) by ultra-high-performance liquid chromatography coupled to ion-trap tandem mass spectrometry (UHPLC-MS/MS). They reported the presence of traces of propranolol, ketoprofen, and diclofenac (beta-blockers, anti-inflammatories, and antipyretics) above the limits set by the EU. Acosta Agudelo et al. (2014) determined the presence of OTCN residues in 149 samples of diaphragmatic muscle from cattle from a slaughterhouse in Caldas, Antioquia (Colombia) using HPLC with diode array detector (HPLC-DAD). According to the findings, 49% of the samples had OTCN residues of which 8% contained amounts higher than the permitted MRLs. Franco et al. (2008) detected the presence of TCN residues in 114 muscle samples from cattle slaughtered in *La Dorada*, Caldas (Colombia) by the ELISA immunoassay technique. The authors reported that 61% of the samples had concentrations higher than 200 µg/kg and did not comply with permissible EU MRLs (100 µg/kg). This demonstrated failures of good practices for the administration of medicines in primary production in the region.

The results allow the possibility of new commercial opportunities since drug residues must be under MRLs established by national and international regulations and the trade agreements increasingly require stricter controls and are essential for international markets. In addition, a food with traces of veterinary drug residues below the maximum allowed limits is considered safe (Udiani et al., 2018), and taking into account that the eating habits of consumers are migrating towards products free of chemical pollutants (Moreno & Lanusse, 2017), people are now looking not only for compliance with the regulatory requirements but also at foods with lower levels of these compounds. Therefore, the levels found below the accepted limits are considered to have a competitive advantage for the country. The development of the livestock sector in the Urabá area is strengthened through the implementation of strategies such as the Antioquia Quality Seal for Bovine Meat Cuts and the promotion of safe, effective, and sustainable systems of production and processing, and marketing of meat and meat products with high-quality standards providing consumer safety.

### 3.2 Heavy metal content

The results of heavy metals in loin and neck cuts of bovine carcasses produced in the Urabá region are listed in Table 3. The samples did not show quantifiable levels for cadmium (Cd), since the levels were below the limits of the used analytical technique (<0.010 mg/kg). Samples showed values between 0.023 and 0.071 mg/kg for lead (Pb) with statistically significant differences (*p* > 0.05) between the different areas of Urabá. The municipalities of the central zone (*Carepa, Chigorodó, Turbo,* and *Apartadó*) showed slightly higher levels than the municipalities of the northern (*Arboletes, San Juan de Urabá, San Pedro de Urabá, Necoclí*) and south (*Mutatá, Murindó*) areas. However, the levels were within the values allowed by Colombian regulations for all cases that establish 0.050 mg/kg and 0.10 mg/kg limits for Cd and Pb Resolution 1382 (2013) (Ministerio de Salud y Protección Social, 2013) and coincide with the European Union established limits for meat products Reg. EU. 37/2010 (EU., 2010). These results indicate that the animals from the Urabá area contained low concentrations of heavy metals Cd and Pb, complying with regulations. This fact is essential for the implementation of the Antioquia Quality Seal strategy for Beef Meat Cuts.

**Table 3:** Cadmium and lead content in meat samples obtained from cattle raised in the Urabá region (Antioquia).

**Municipality**	**Number of samples**	**Lead (mg/kg)**	**Cadmium (mg/kg)**
*Arboletes*	12	0.026 ± 0.010	<0.010
*San Juan de Urabá*	10	0.037 ± 0.011	<0.010
*Necoclí*	8	0.023 ± 0.003	<0.010
*San Pedro de Urabá*	9	0.058 ± 0.009	<0.010
*Apartadó*	12	0.061 ± 0.024	<0.010
*Carepa*	8	0.051 ± 0.022	<0.010
*Chigorodó*	6	0.036 ± 0.012	<0.010
*Turbo*	5	0.071 ± 0.028	<0.010
*Mutatá*	2	0.044 ± 0.024	<0.010
*Murindó*	3	0.027 ± 0.010	<0.010

Excessive intake of Cd and Pb are characterized by nausea, vomiting, abdominal pain, diarrhea, headache, anemia, and liver disorders (Johri et al., 2010; Shiri et al., 2007), and other toxic effects on the gastrointestinal tract, renal, immune, reproductive, cardiovascular, peripheral and central nervous systems (Andjelkovic et al., 2019). Lead interferes with several biochemical processes in the body by binding to sulfhydryl and other nucleophilic functional groups causing inhibition of several enzymes and changes in calcium/vitamin D metabolism. Lead also contributes to oxidative stress within the body and inhibits the body's ability to make hemoglobin by interfering with several enzymatic steps in the heme pathway, thus, contributing to the anemia that develops in chronic lead poisoning (Gupta & Wong Wai Leong, 2011). Mammals, birds, and reptiles have all been found to develop lead poisoning. Lead affects cognitive development and intellectual performance in children and increases blood pressure and cardiovascular disease in adults (Cho et al., 2010; Nigra et al., 2016).

Because of these important adverse effects, different researchers have conducted similar studies. Lopez Alonso et al. (2000) determined the levels of traces of some metals including Cd and Pb, in muscle, liver, and kidney of calves and cows slaughtered in Galicia (Spain). The results showed a content of Cd in the liver and kidney that was 3 and 6.5 times higher in cows than in calves. In muscle, the content was similar since 7% of cows and 0.2% of calves showed higher levels than the limits allowed by the EU. The concentration of Pb in muscle was 88% higher than in calves, showing that the concentrations increased with age, but they were still below the maximum limits. Zeinali et al. (2019) analyzed 102 samples of beef muscle, liver, and kidney taken randomly in southeastern Iran, and they found higher levels of Cd and Pb in the liver and kidney than in muscle, where the levels did not exceed the MRLs. For that study, an inductively coupled plasma-optic emission spectroscopy (ICP-OES) was used for quantification. Madero and Marrugo (2011) evaluated the presence of heavy metals including Cd and Pb in samples of bovine liver and pectoral muscle in Córdoba (Colombia). These metals were quantified by the anodic stripping voltammetry method, and they found that 96% of the samples showed metal levels below the permissible limits in the EU and Mexico. Valladares-Carranza et al., 2015, analyzed the concentration of Pb in cattle slaughtered in Toluca (Mexico) employing atomic absorption spectrophotometry. They reported that 7.10% of the samples showed higher values than the MRLs (1.0 mg/kg). However, considering the range of results from 0.3 to 1.9 mg/kg, none of the samples adjust to the standards established by the EU. González-Weller et al. (2006) evaluated the content of Pb and Cd in various meats (chicken, pork, beef, lamb, and turkey) and meat products consumed in Tenerife (Spain) and found high variability in the results. Lead and cadmium were measured by graphite furnace atomic absorption spectrometry (GFAAS). Although the levels were below the limits established by the EU, the authors established that this variability is considered normal since the sources of metals are numerous, and in addition, the concentrations of Pb and Cd also depend on environmental conditions and food production methods.

## Conclusions

The presence of veterinary drug residues and heavy metals Cd and Pb, in the different beef samples from the Urabá region in Antioquia, Colombia, were below the maximum residual limits (MRL) of national and international regulations established by the European Union. Given that the use of veterinary drugs can treat bovine diseases, their application appropriately and safely through the implementation of good practices that ensure requirements and promotes the development of the livestock industry. By continuing the model implemented for agronomic and veterinary management carried out in the Urabá area through monitoring and control of good agricultural and livestock practices and complying with not only national but also international regulations allows entering new markets and achieving greater competitiveness. This will strengthen local capacities in livestock production and will generate new opportunities of economic growth for the region.

## Ethical Statements

1) This material is the authors' own original work, which has not been previously published elsewhere.
2) The paper is not currently being considered for publication elsewhere.
3) The paper reflects the authors' own research and analysis in a truthful and complete manner.
4) The paper properly credits the meaningful contributions of co-authors and co-researchers.
5) The results are appropriately placed in the context of prior and existing research.
6) All sources used are properly disclosed.
7) All authors have been personally and actively involved in substantial work leading to the paper.

## Declaration of Competing Interest

The authors declare that they have no known competing financial interests or personal relationships that could have appeared to influence the work reported in this paper.

## Appendix Supplementary materials

Image, application 1
