# Supplementary material for: Residues of veterinary drugs and heavy metals in bovine meat from Urabá (Antioquia, Colombia), a promising step forward towards international commercialization
Source: Vet Anim Sci. 2021 Aug 4;13:100192. doi: 10.1016/j.vas.2021.100192 (PMC8363876; doi:10.1016/j.vas.2021.100192)
Supplement: Supplementary file 1 [file mmc1.docx]

**Residues of veterinary drugs and heavy metals in bovine meat from Urabá (Antioquia, Colombia), a promising step forward towards international commercialization**

**Supplementary information S1: Anatomical description of the meat cuts**

**Neck cut:** Initiate from the atlas to the third thoracic vertebra, the cut was traced along the cranial edge of the scapula, descending distally, looking for the shoulder joint (scapulo-humeral), carving against the vertebrae and removing the complete cut, muscles involved: cervical portion trapezius, omotransverse, brachiocephalic, external brain, external thyrohyoid, rhomboids, splenium, serratus, rectus neck.

**Loin cut:** from the third to the thirteenth thoracic vertebra, a deep dissection is performed up to the vertebral processes and against the corresponding ribs in their joint, muscles involved: Length of the back, Great dorsal, Length of the loin.
